# Impact of motivational interviewing-based training in screening, brief intervention, and referral to treatment on residents’ self-reported attitudes and behaviors

**DOI:** 10.1186/1940-0640-8-S1-A71

**Published:** 2013-09-04

**Authors:** J Paul Seale, Denice C Clark, Jason Dhabliwala, David Miller, Hunter Woodall, Sylvia Shellenberger, J Aaron Johnson

**Affiliations:** 1 Department of Family Medicine, Medical Center of Central Georgia, Macon, GA, USA; 2 Department of Family Medicine, Mercer University School of Medicine, Macon, GA, USA; 3 Department of Internal Medicine, Wake Forest University Baptist Medical Center, Winston-Salem, NC, USA; 4 Med Health Family Medicine Residency, Anderson, SC, USA

## Introduction

Many medical residents now receive training in screening, brief intervention, and referral to treatment (SBIRT) for alcohol and drugs. Clinician attitudes have been shown to impact SBIRT-related behaviors. Little research has explored the impact of SBIRT training on clinicians’ attitudes.

## Objective

To determine whether SBIRT training impacts resident physicians’ alcohol-related attitudes and self-reported SBIRT activities.

## Methods

Residents participating in SBIRT training in 4 U.S. primary care residency programs were surveyed during Months 0, 12, and 24. Interventions included training faculty site coordinators, providing residents 6 hours of motivational interviewing (MI)-based SBIRT curriculum per year, and implementing SBI protocols in residency clinics. Impact was assessed using the Short Alcohol and Alcohol Problems Perception Questionnaire (SAAPPQ) and residents’ self-reported frequency of alcohol screening, brief interventions (BIs) and use of specific BI components. Analyses assessed changes over time in SAAPPQ total and subscale scores, changes in BI performance and use of BI components, and the relationship between attitude scores and frequency of performing BIs and specific BI components.

## Results

Residents’ scores on the role adequacy subscale of the SAAPPQ increased significantly between baseline and 12 month survey (p<.001). This increase was sustained at 24 months (p<.001). Other subscales and overall SAAPPQ scores did not change. Chi-square tests showed significant increases over time in the frequency of screening at chronic care visits (p=.02) and in use of BI elements including: providing feedback (p=.003), advising to cut down/quit (p<.001), using MI techniques to motivate (p<.001), negotiating a plan (p=.01), and emphasizing patient strengths (p<.001). Regression analyses found a significant positive relationship between SAAPPQ scores and both frequency of performing BIs and the use of appropriate BI elements.

## Conclusion

Twelve hours of SBIRT training, when accompanied by faculty training and clinic SBI implementation protocols, has a modest positive impact on resident attitudes and SBIRT behaviors.

